# Measuring Correlates of Mental Workload During Simulated Driving Using cEEGrid Electrodes: A Test–Retest Reliability Analysis

**DOI:** 10.3389/fnrgo.2021.729197

**Published:** 2021-09-14

**Authors:** Stephan Getzmann, Julian E. Reiser, Melanie Karthaus, Georg Rudinger, Edmund Wascher

**Affiliations:** ^1^IfADo - Leibniz Research Centre for Working Environment and Human Factors, Dortmund, Germany; ^2^Uzbonn - Society for Empirical Social Research and Evaluation, Bonn, Germany

**Keywords:** EEG, driving, mental work load, cEEGrids, test-retest reliability

## Abstract

The EEG reflects mental processes, especially modulations in the alpha and theta frequency bands are associated with attention and the allocation of mental resources. EEG has also been used to study mental processes while driving, both in real environments and in virtual reality. However, conventional EEG methods are of limited use outside of controlled laboratory settings. While modern EEG technologies offer hardly any restrictions for the user, they often still have limitations in measurement reliability. We recently showed that low-density EEG methods using film-based round the ear electrodes (cEEGrids) are well-suited to map mental processes while driving a car in a driving simulator. In the present follow-up study, we explored aspects of ecological and internal validity of the cEEGrid measurements. We analyzed longitudinal data of 127 adults, who drove the same driving course in a virtual environment twice at intervals of 12–15 months while the EEG was recorded. Modulations in the alpha and theta frequency bands as well as within behavioral parameters (driving speed and steering wheel angular velocity) which were highly consistent over the two measurement time points were found to reflect the complexity of the driving task. At the intraindividual level, small to moderate (albeit significant) correlations were observed in about 2/3 of the participants, while other participants showed significant deviations between the two measurements. Thus, the test-retest reliability at the intra-individual level was rather low and challenges the value of the application for diagnostic purposes. However, across all participants the reliability and ecological validity of cEEGrid electrodes were satisfactory in the context of driving-related parameters.

## Introduction

Neurophysiological research methods have a long tradition of deriving mental processes both under laboratory conditions and in real-life environments. While in the first case a high degree of experimental control and reliability of measurements is can be assumed, measurements of neurophysiological parameters in the field (still) represent a challenge, but also an opportunity toward a higher ecological validity (Engel et al., 2013; Parada, 2018; Parada and Rossi, 2020). Especially with regard to EEG, the development of modern recording methods and analysis routines has opened up completely new possibilities to map the work of the brain under real conditions (for a recent review, Wascher et al., 2021). In two recent studies, for example, we showed that mental workload during the processing of cognitive tasks while walking on differently challenging courses was not only reflected in performance measures, but that it was also associated with modulations in brain activity (Reiser et al., 2019, 2020). While both studies clearly demonstrated the usability of EEG measurements under out-of-laboratory everyday conditions, conventional electrode caps have been used here, which offer a good prerequisite for EEG recording, but are unfavorable in real-life environments for many reasons: they are conspicuous, time-consuming to apply, restrict the user's mobility, and are of limited use when high ecological validity is important – especially when possible influence of the measurement method on the measurement results should be minimized (e.g., Sterr et al., 2018; Mikkelsen et al., 2019).

Alternative solutions are provided by new recording technologies. The use of dry electrodes, for example, is such a technology, which has proven to be very reliable, but easier to apply and wear compared to conventional wet electrodes (Di Flumeri et al., 2019). Even more inconspicuous is the cEEGrid system, in which the EEG is recorded by only a few film-based round the ear electrodes. The cEEGrids technology not only avoids restrictions arising from conventional electrode setups (Symeonidou et al., 2018), but is also easier and faster to apply than conventional multichannel Cap-EEG. At the same time, they offer a sufficient signal quality and allow for valid and reliable measurements (Mirkovic et al., 2016; Bleichner and Debener, 2017). Previous research has shown, for example, that it is possible to derive neurophysiological correlates of cognitive processes from the oscillatory brain activity recorded via cEEGrid electrodes both in an auditory oddball task (Debener et al., 2015) and a visual Simon task (Pacharra et al., 2017).

The good practicability of the cEEGrids technology was only recently demonstrated in a large-scale study on driving abilities of seniors, in which older adults drove an ~1-h close-to-reality driving simulator course, consisting of different road sections with various challenges for the driver (Wascher et al., 2019). Using behavioral (driving speed, steering wheel angular velocity) and neurophysiological measures (EEG oscillatory power in the theta and alpha band frequencies), it was possible to estimate mental workload while driving, based only on characteristics of the driving situation. They found that with increasing track difficulty the steering angular velocity increased while driving speed decreased. A similar pattern was found on the electrophysiological level, whereas relative theta power increased and relative alpha power decreased. Finally, using a track-frequency analysis, it was possible to map modulations in EEG spectral power to the difficulty of the traffic situation, which highly corresponded with a priori expert ratings. This highlights the connection of behavioral and electrophysiological measures, as the findings are in line with the assumption that, firstly, reduced alpha power is a correlate of increased mental workload (Wascher et al., 2016) and attentional engagement (Pattyn et al., 2008), and, secondly, increased theta power is related to mental processing demands (Lal and Craig, 2001; Borghini et al., 2014) and associated with higher workload (Wilson and Hankins, 1994; Gevins et al., 1997) or task engagement (Yamada, 1998; Onton et al., 2005). However, the cognitive processes represented by alpha and theta activity cannot be considered separately. Especially in natural environments, for example, when driving a car (Di Flumeri et al., 2018) and when multi-tasking is required (Puma et al., 2018), numerous subtasks have to be performed, which are represented differently in oscillatory brain activity. It has been proposed that visual processing, information-gathering, and early attention allocation seems to be represented more by alpha activity, while higher cognitive processes such as integration of information, problem solving, and executive functions seem to be represented more by theta activity (Berka et al., 2007). This is also reflected in the topography, with alpha activity typically derived over parietal and theta activity over fronto-central areas (e.g., Wang et al., 2018; for review, Klimesch, 1999). Accordingly, by combining driving parameters and oscillatory activity in the alpha and theta frequency bands derived over parietal and frontal areas, respectively, it has recently been demonstrated that the current workload of a driver can reliably be determined using a mobile EEG system (Islam et al., 2020). Taken together, both measures demonstrated the flexible allocation of cognitive resources depending on the route section and difficulty (Borghini et al., 2014; Karthaus et al., 2018; for review, Lohani et al., 2019).

Results like these are overall promising, but lead toward a still unanswered question: to what extent are these EEG measurements reliable? This arising question of EEG test-retest reliability is nothing new, as studies on resting-state EEG proved that the normal EEG can be treated as an intraindividually rather stable trait (e.g., Gasser et al., 1985; Van Albada et al., 2007; Angelidis et al., 2016), with test-retest reliabilities in healthy adults typically exceeding 0.80 over intervals of more than 1 year (Hatz et al., 2015). Adding to this, task-related EEG which maps changes in cognitive states related to, for example, task difficulty was also found to have a high test-retest reliability. An exemplary study was conducted by McEvoy et al. (2000), in which subjects performed cognitive tasks at intervals of 7 days, resulting in high intraindividual correlations in oscillatory brain activity in the theta and alpha frequency bands. Comparably high reliabilities were also found in other works (e.g., Fernández et al., 1993; Fallgatter et al., 2002; Näpflin et al., 2008). In the context of driving, a study on the reproducibility of EEG modulations as consequence of driver fatigue showed high test-retest reliability as well (Lal and Craig, 2005). However, transient fluctuations in mental states like alertness and vigilance are hard to control especially under less structured experimental conditions and have typically been associated with reduced test-retest reliabilities – a pattern typically found in natural environments (Fernández et al., 1993). For mobile EEG systems, only few findings are available so far. A study in which the test-retest reliability of a single-channel, wireless EEG system was tested in healthy individuals showed reduced, but still satisfactory reliabilities over short (1-day) and longer (1-week and 1-month) retest-intervals, with Intra-Class Correlations for a group of older adults ranging between 0.51 and 0.89 in an eyes-open condition (Rogers et al., 2016). A study on cEEGrids demonstrated a sufficient test-retest reliability when measuring resting-state and task-related EEG in an auditory oddball paradigm over many hours (Debener et al., 2015).

The aim of the present study was to evaluate the test-retest reliability of the cEEGrid technology under less favorable recording conditions over an even longer time interval. For this purpose, the data of the first measurement point of our driving study presented in Wascher et al. (2019) were compared with those of the second measurement more than 1 year later. All data analyzed here were taken from a (still ongoing) large-scale investigation of the driving abilities of older adults aged between 67 and 76 years, which is designed as a longitudinal study with the same individuals being tested several times at intervals of 12–15 months. In addition to several neuropsychological tests, the project also comprises a simulated driving test during which the EEG is recorded using cEEGrid technology. The comparison between the two time points of measurement was performed on the behavioral (i.e., driving speed and steering wheel angular velocity) and EEG data (alpha and theta power) as well as their dependencies on the characteristics of the driving route. In addition, it was assessed to what extent interindividual differences could be replicated regarding the allocation of mental resources as a function of workload. Thus, while our former study demonstrated that task-related modulations of driving behavior and EEG—previously found in controlled lab settings—are also observable in a naturalistic driving simulation and cEEGrids measurements, now we focused on the following questions: (1) How have the performance parameters assessed during the driving course (i.e., driving speed and steering wheel angular velocity) changed compared to the first measurement point? (2) Can the previously found dependence of relative alpha and theta power on track difficulty be replicated at a between-subject level? (3) How strong is the intraindividual correspondence of the oscillatory measures in dependence on the track difficulty?

## Methods

### Participants

All participants were part of a large-scale longitudinal investigation of the driving abilities of older adults which started in 2016. One hundred twenty-seven participants took also part in both measurement time points, completed the required driving distance twice and provided a sufficient data quality in the EEG (see below). These 127 participants (mean age 72.2 years, age range 68–77 years; 22.0 % female) all had a valid driving license and reported to be experienced drivers with an average annual mileage between 5,000 and 10,000 km/year. They had normal or corrected to normal vision and reported an overall good health status. They completed a battery of neuropsychological tests which will not be reported here. Before starting the experiment, all participants provided written informed consent. The study was approved by the local ethics committee of the Leibniz Research Centre for Working Environment and Human Factors.

### Task and Procedure

The task and the experimental procedure were exactly the same for measurement points 1 and 2 (MP1 and MP2). Between MP1 and MP2, there was an average of 398.17 days (minimum 350, maximum 580, SD 37.79; about 13 months). The test procedure and data analysis have been described in detail in Wascher et al. (2019). In brief: After completing various questionnaires and performing a battery of neuropsychological followed by a vision test, the participants completed a pre-test drive lasting about 15 min. The driving route of the pre-test drive was not part of the actual test drive and intended to familiarize the drivers with the characteristics of the vehicle, its steering and braking behavior, and the static driving simulator (ST Sim, St Software B.V. Groningen, NL). Then the cEEGrid electrodes were attached and the participants completed a driving course which resembled a regular German driving test consisting of four different road sections: a section of state road with several intersections, roundabouts, and a foggy passage (SR1) was followed by a longer freeway section including several roadwork sites and a freeway parking area had to be passed (FW). This was followed by another section of state road with several left and right turn intersections (SR2), before the drivers entered the city where traffic lights, pedestrians, and cyclists had to be attended to (CT). Acoustic (verbal) and visual navigation information guided the drivers through the ~37-km driving course. Given that not all participants finished the complete course, only the first 30 km were analyzed here.

In order to test how the mental workload of the driver was modulated by the characteristics of the driving route, the driving scenario was a priori subdivided into three driving profiles, being either simple (undisturbed ride on a free route), complex (junctions with turning, roundabouts, left turns, traffic lights, motorway entrances and exits), or interactive (interactions with other traffic participants, like overtaking or driving behind a vehicle ahead). In total, sections of simple, complex, and interactive driving profiles comprised ~13, 8, and 9 km, respectively. These driving profiles were classified by an expert according to their assumed mental demands as of low, medium, and high task load (cf. Pauzié, 2008; Rahman et al., 2017). It should be noted, however, that this subdivision was done across all road sections (i.e., state road, freeway, and city sections), since the proportion of different route profiles was distributed rather unevenly across the road sections. Since the driving distance had to be limited to a reasonable level (also in view of the background of the study and the age of the participants), the data basis did not appear to be sufficient for a more fine-grained differentiation.

### Data Recording and Processing

**Figure 1:**
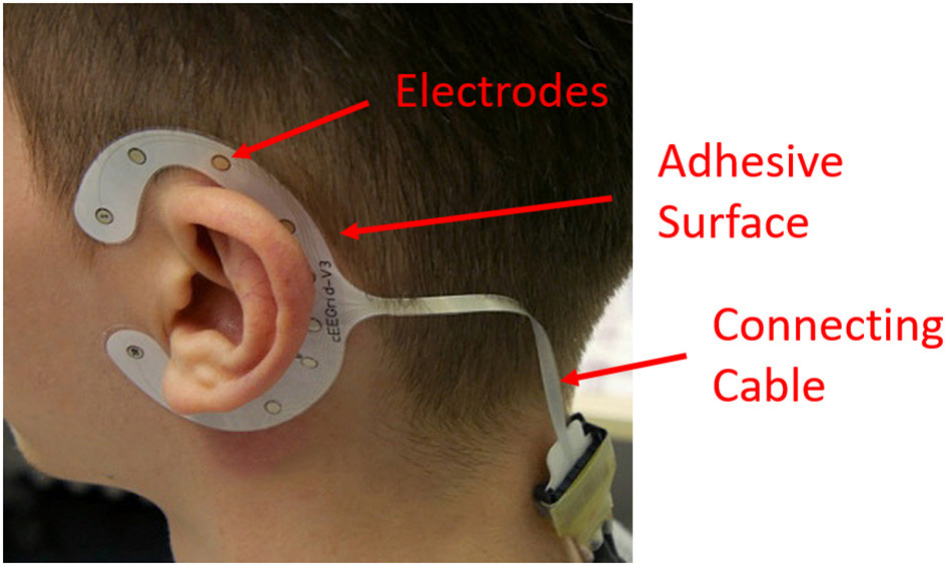
cEEGrids technology, consisting of C-shaped electrode arrays with 10 electrodes placed around the participant's left and right ears (photo: IfADo).

EEG was recorded using cEEGrids, consisting of flex-printed, C-shaped electrode arrays with 10 silver printed electrodes (Debener et al., 2015; Bleichner et al., 2016; Mirkovic et al., 2016; Pacharra et al., 2017). The cEEGrids are positioned around the participant's left and right ear using an adhesive surface (Figure 1). In contrast to conventional electrode setups, cEEGrids are barely visible, comfortable to wear, require only a small amount of electrode gel, and are therefore fast and easy to apply and remove. The cEEGrids were connected to a QuickAmp DC-amplifier with an on-line low-pass filtering at 280 Hz. Data were sampled at 1 kHz with a resolution of 24 bits. The two electrodes in the middle of the right cEEGrid served as ground and online reference respectively (R4a, R4b). EEG data were stored together with the driving simulator data from which driving speed and steering wheel angular velocity were derived offline. Driving speed was defined as the distance (in meters) traveled per time (in seconds) over a distance of 10 m and converted in kilometers per hour (km/h). Steering wheel angular velocity was defined as the angular speed at which the drivers turned the steering wheel, averaged over a distance of 10 m and converted in degrees per second (deg/s). In general, steering wheel angular velocity is considered an indicator of task load while driving (e.g., Antin et al., 1990; Verwey and Veltman, 1996).

The EEG analysis procedure is described in detail in Wascher et al. (2019) and is therefore only outlined briefly here. Firstly, data were checked for integrity, so that data sets with either incomplete driving distance or corrupt transmission of simulator data into the EEG recording files were discarded. After resampling to 200 Hz and band-pass filtering (1–40 Hz) of the EEG and simulator data, single EEG channels were checked for integrity by using the EEGLAB implemented rej_channel function (normed data; criterion: 4 standard deviations) to detect and discard faulty channels. Only datasets with intact reference channels after channel rejection were kept for further analyses. They were re-referenced to the average of L4b and R4b and entered into the artifact subspace reconstruction (ASR) procedure (Mullen et al., 2014, 2017). ASR is a component-based method and was proven in a number of studies (e.g., Plechawska-Wojcik et al., 2019) including a driving simulator study (Chang et al., 2019) to be highly effective in automatic filtering transient or large-amplitude artifacts (like produced by eye blinks and eye movements) from EEG data. Followingly, a time frequency decomposition was performed on each channel by convolving the data with complex Morlet wavelets. Spectral power estimates were calculated as the squared absolute values of the complex convolution result and were averaged across channels. Finally, participants with total EEG power that deviated by more than 3 standard deviations from the median were discarded and the complete data set was excluded. In total, the 127 participants (described in section Participants) who had complete data sets at both measurement points were included into the further analysis.

### Data Analysis

We conducted two different approaches to assess the retest reliability of the EEG data, first a *task-load related analysis*, investigating whether the EEG measures at both measurement points depended on the driving profile in the same way, and second an (intra-individual) *correlational analysis*, comparing the EEG measures along the route at MP1 and MP2 separately for each subject. In addition to the spectral power in theta (3–6 Hz) and alpha (7–10 Hz) frequency bands, behavioral data (driving speed and steering wheel angle velocity) were analyzed to test whether behavioral results reflect the same pattern as the EEG results. It should be noted that we chose a lower than typical frequency range for determining alpha activity. The reason for this is the shift in alpha activity toward lower frequencies that is often observed with increasing age (e.g., Van Albada et al., 2010; Chiang et al., 2011). In our earlier analysis, we also measured a mean alpha frequency of <9 Hz and therefore chose the frequency range of 7–10 Hz (Wascher et al., 2019). For this reason, and also for reasons of better comparability with our previous study, we have maintained this frequency range here as well.

In the task-load related analysis, behavioral and EEG data were averaged across the driving course, separately for simple, complex, and interactive driving profiles, and mean values were entered into 2 × 3 ANOVAs with measurement point (MP1, MP2) and driving profile (simple, complex, interactive) as within-subjects factors. Effect size estimates (adj $\eta_{\text{p}}^{2}$) are reported according to Mordkoff (2019). As in our former study, not only raw power values of alpha and theta activity were analyzed, but also relative power values, representing the percentage of the power in a given frequency band relative to the total power. We therefore calculated the contribution of each frequency to the overall signal by applying a vector normalization across all frequencies for each time point. The result were the so-called alpha and theta *fractions*. The idea behind this normalization is that high power and high variance in oscillatory activity across all frequency ranges often masks effects in the alpha and theta regions, which may become more prominent by forming the relative power values. Thus, there is evidence that relative power fluctuations are more related to experimental effects than absolute power fluctuations (Klimesch, 1999; Kilner et al., 2005; Labounek et al., 2015).

For the correlational analysis, we conducted the track-frequency analysis (as detailed in Wascher et al., 2019), in which the time period of the EEG recording was mapped onto the 30-km driving route using 43 predefined landmarks for each participant. The landmarks consisted of defined route points to which a trigger was written into the EEG recording as soon as the vehicle passed this point. For the sections between the landmarks, the waypoints were estimated from the current speed of the vehicle at that point. Thus, we achieved a temporal-spatial assignment, in which each time point of the EEG measurement was assigned to a track section by stretching and compressing the EEG data in the temporal domain. For the track-frequency analysis, 3,000 10-meter track segments were generated, covering the entire 30-km driving route. To determine the alpha and theta power along the track, first a time-frequency analysis was performed over all time points. Based on this analysis, the mean power fraction for alpha and theta power was calculated for each of the 10-meter track segments, then z-transformed across all the 3,000 data points and low-pass filtered by a ± 40 m moving average. The 95% confidence intervals were calculated and are shown in **Figure 4** for MP1 and MP2. In order to determine the relationships of oscillatory power measured at the two measurement points on an intraindividual level, correlations between MP1 and MP2 were computed across the entire 30-km driving course. That is, Pearson's r correlations between the alpha and theta power values measured at MP1 and MP2 were computed across the 3,000 10-meter track segments for each participant. Associations between the two measurement points were regarded as weak, moderate, or high for correlation coefficients of 0.10, 0.30, or 0.50 or larger, respectively, according to the interpretation of effect sizes proposed by Cohen (2013). Since effects of the driving course should rather appear on relative (than on absolute) power values (see above), the correlation analysis was exclusively performed for alpha and theta fractions.

The track-frequency analysis was completed by a simple classifying algorithm intended to estimate the track-specific task load based on the EEG data. Here, it was assumed that high theta activity is associated with increased mental effort and high alpha activity with reduced attentional allocation. Therefore, as in our previous analysis (Wascher et al., 2019), the algorithm tested theta and alpha fraction against each other using a paired-sample *t*-test for each data point (i.e., for each 10-meter track segment), and assigned *low task load* to track segments with significantly higher alpha than theta fraction, and *high task load* to segments with significantly higher theta than alpha fraction. If theta and alpha fraction did not differ significantly, a *median task load* was assigned to this track segment. This classification procedure was performed equally for MP1 and MP2, and it was determined how many road sections were rated as equally difficult at both measurement times (suggesting a reliable estimation of task load from the EEG) or were rated as easier or more difficult in MP1 and MP2. Finally, for each participant the correlation of EEG-based task load estimates at MP1 and MP2 was computed across the entire 30-km driving course (i.e., across the each 10-meter track segment), using Pearson's r correlations.

## Results

### Behavior

**Figure 2:**
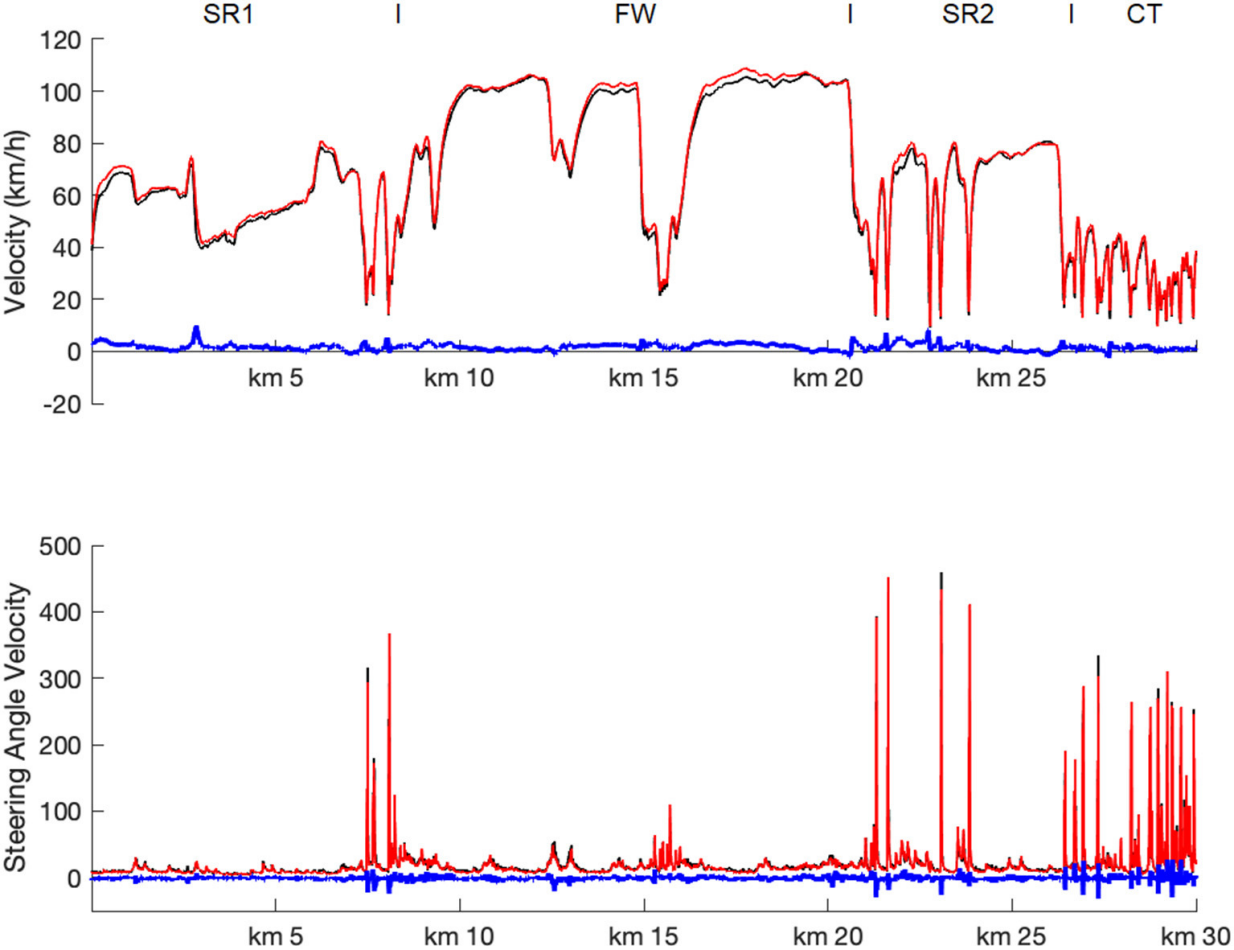
Track-based analyses of driving parameter. Mean driving speed (upper row) and steering wheel angular velocity (lower row) as function of driving route, shown separately for MP1 (black), MP2 (red), and MP1 – MP2 differences (blue). Note that for each time point individual values of each participant were assigned to fix waypoints and then averaged 10-meter wise. SR1, first state road; FW, freeway; SR2, second state road; CT, city traffic.

The track-based analyses of the driving parameters showed that both driving speed and steering wheel angular velocity profoundly varied along the driving course (Figure 2). In particular, while the freeway section (FW) was characterized by high driving speed and low steering angular velocity (apart from passing through a freeway parking area at kilometer 15), the second state road section (SR2) and especially the city traffic drive (CT) were characterized by lower and highly varying driving speed as well as increased and higher steering angular velocities. More importantly, however, it is to notice that the driving speed increased overall, while the steering wheel angular velocity decreased at MP2 relative to MP1. Also, the mean drive time for the entire course went from 51.6 min (SD 9.0) to 46.7 min (SD 8.3), *t*_(126)_ = 5.65, *p* < 0.001.

**Figure 3:**
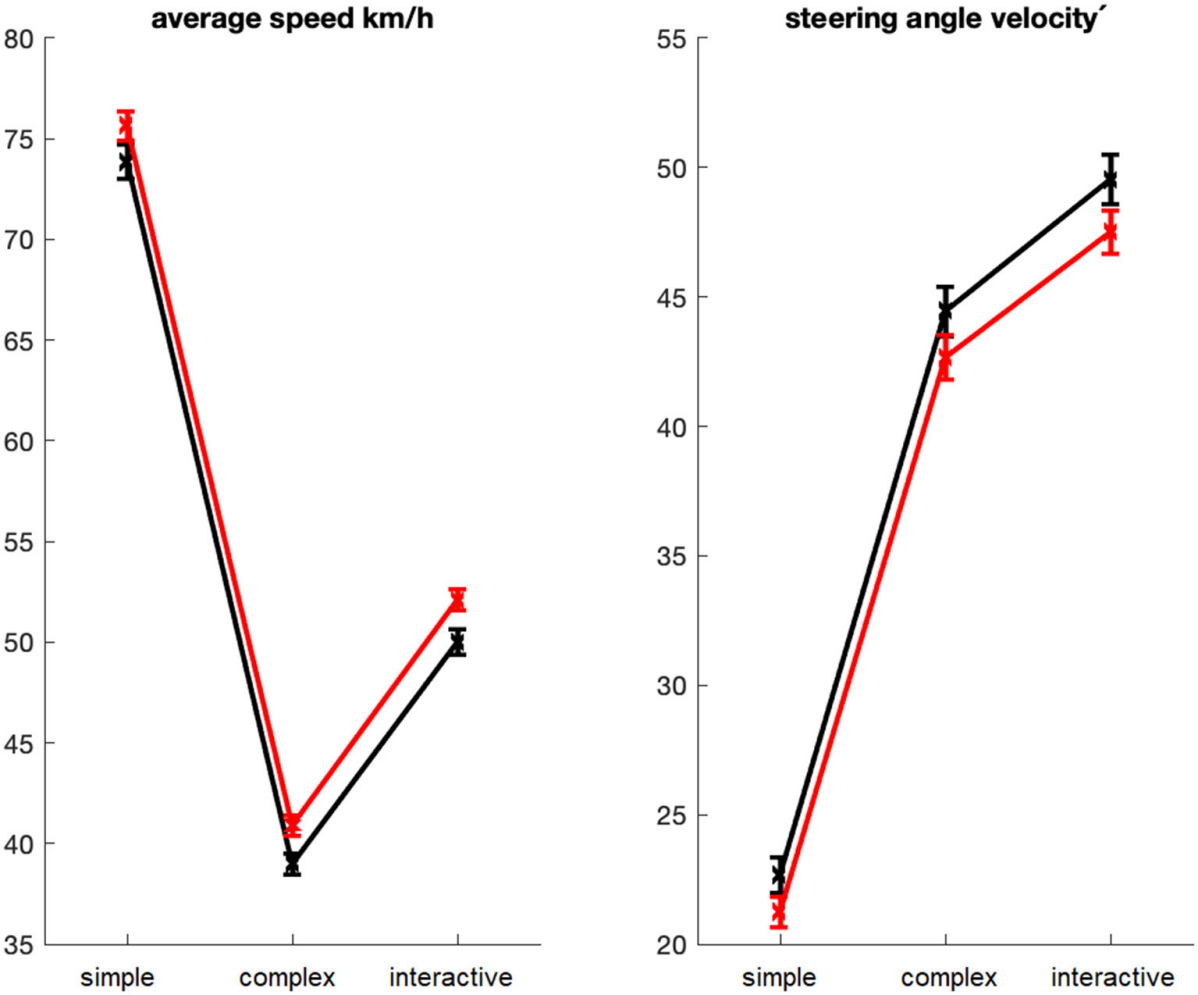
Task-load related analysis of driving parameters. Average driving speed (left) and steering angle velocity (right) as function of driving profile (simple, complex, interactive), shown separately for MP1 (black) and MP2 (red). Error bars indicate standard errors.

These differences were even more evident in the task-load related analysis, analyzing the driving parameters separately for passages with simple, complex, and interactive driving profiles (Figure 3). The mean driving speed significantly increased from MP1 to MP2, *F*_(1, 126)_ = 25.60, *p* < 0.001, adj $\eta_{\text{p}}^{2}$ = 0.162, while the mean steering wheel angular velocity decreased, *F*_(1, 126)_ = 20.15, *p* < 0.001, adj $\eta_{\text{p}}^{2}$ = 0.131. There were no interactions of measurement time and driving profile, neither for driving speed, *F*_(2, 252)_ = 0.35, *p* = 0.71, adj $\eta_{\text{p}}^{2}$ = 0.005, nor for steering wheel angular velocity, *F*_(2, 252)_ = 1.36, *p* = 0.26, adj $\eta_{\text{p}}^{2}$ = 0.003, indicating that the effects of driving profile on driving speed, *F*_(2, 252)_ = 4,402.95, *p* < 0.001, adj $\eta_{\text{p}}^{2}$ = 0.972, and steering angular velocity, *F*_(2, 252)_ = 2,118.03, *p* < 0.001, adj $\eta_{\text{p}}^{2}$ = 0.944, did not depend on measurement time. Thus, the participants drove at highest speed in simple passages and significantly reduced the speed in complex passages, *F*_(1, 126)_ = 5,488.40, *p* < 0.001, adj $\eta_{\text{p}}^{2}$ = 0.977. Relative to complex passages, they also drove faster when there were interactions with other road users, *F*_(1, 126)_ = 1,369.34, *p* < 0.001, adj $\eta_{\text{p}}^{2}$ = 0.915. The steering angular velocity increased from simple to complex passages, *F*_(1, 126)_ = 5,919.92, *p* < 0.001, adj $\eta_{\text{p}}^{2}$ = 0.979, and further from complex to interactive passages, *F*_(1, 126)_ = 169.92, *p* < 0.001, adj $\eta_{\text{p}}^{2}$ = 0.571.

### Alpha and Theta Power Analysis

**Figure 4:**
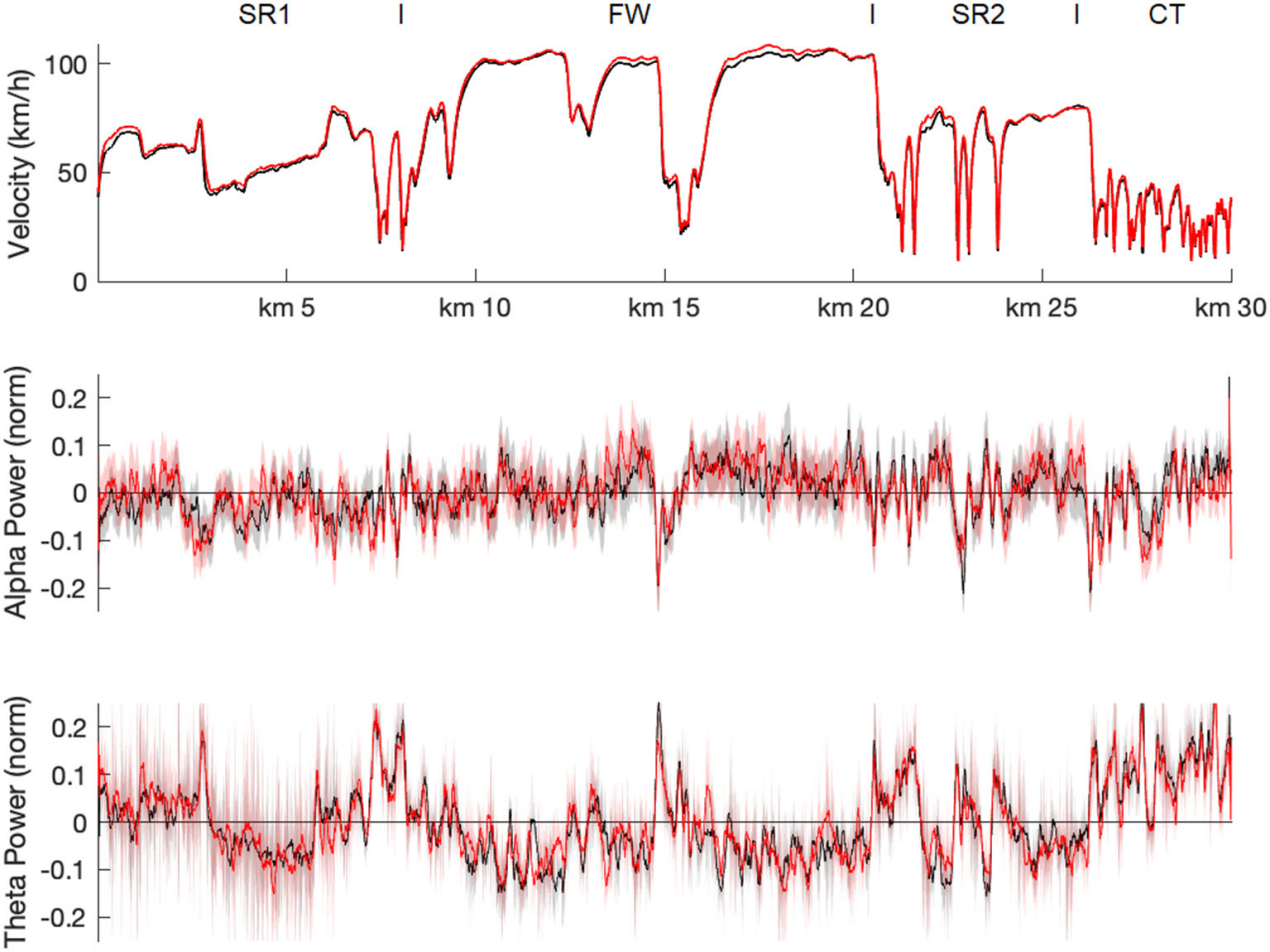
Track-based analyses of EEG parameter. Mean z-transformed alpha (middle row) and theta (lower row) power fractions as function of driving route for MP1 (black) and MP2 (red), shaded by their 95% confidence intervals. For comparison, mean driving speed (upper row) is also shown. Note that for each time point individual alpha and theta values of each participant were assigned to fix waypoints and then averaged separately for 10-meter track segments. SR1, first state road; FW, freeway; SR2, second state road; CT, city traffic.

The track-based analysis of brain oscillatory power demonstrated that both alpha and theta power fractions varied substantially over the driving route (Figure 4): Phases of high alpha fraction alternated with short sections in which alpha fraction was strongly reduced. For example, the freeway passage was characterized by high alpha fraction values, while these were reduced at the beginning of the fog passage at kilometer 3, when driving through the freeway parking area at kilometer 15, and during city driving after kilometer 27. The theta values, on the other hand, showed a rather inverse pattern.

**Figure 5:**
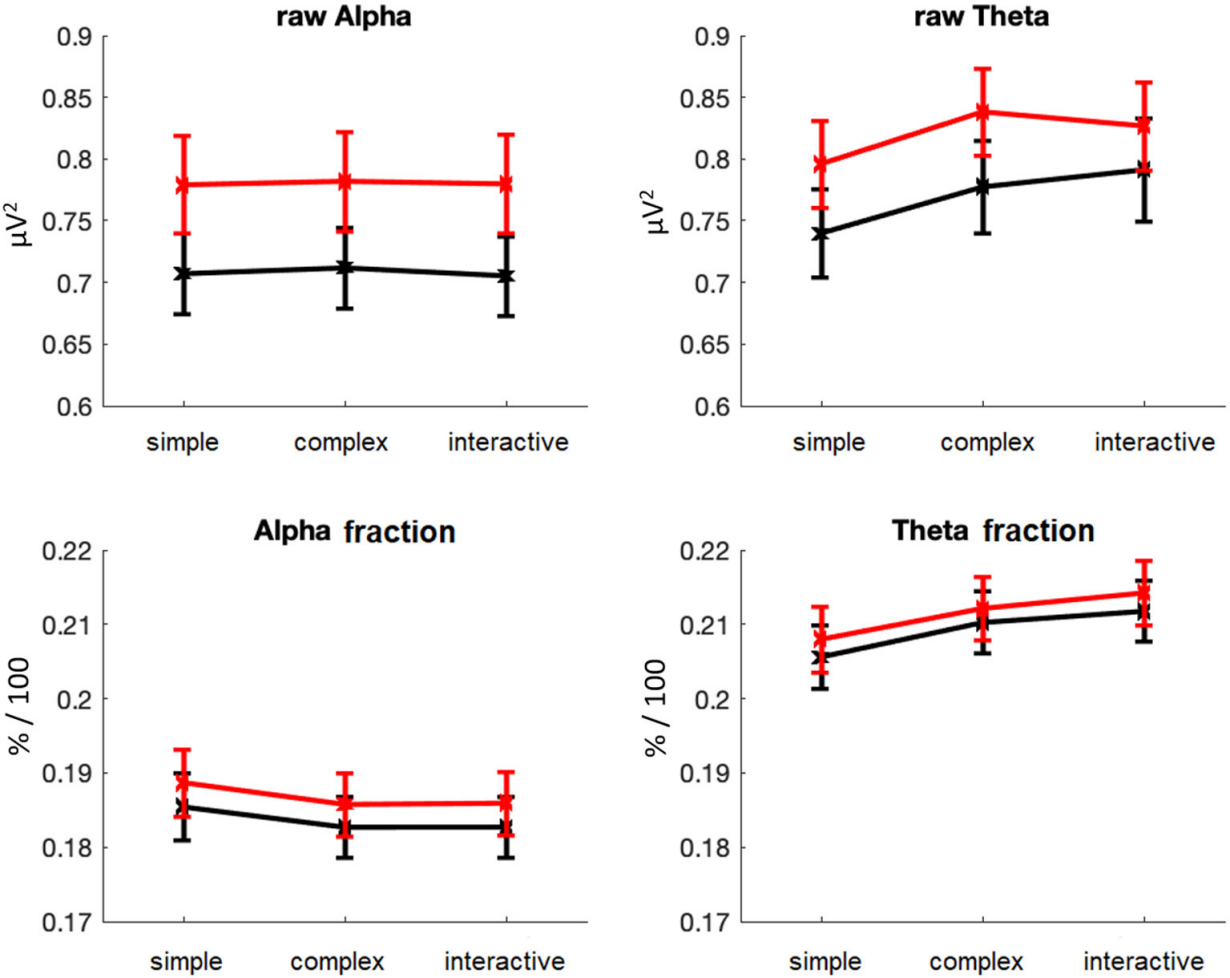
Task-load related analysis of EEG parameters. Raw (upper row) and fractional (lower row) average power in the alpha (left) and theta (right) frequency bands as function of driving profile (simple, complex, interactive), shown separately for MP1 (black) and MP2 (red). Error bars indicate standard errors.

The task-load related analysis indicated that raw alpha power significantly increased from MP1 to MP2, *F*_(1, 126)_ = 10.02, *p* < 0.005, adj $\eta_{\text{p}}^{2}$ = 0.066, while differences for raw theta power and alpha and theta fraction power were not significant, all *F*s < 2.13, all *p*s > 0.14 (Figure 5). There were effects of driving profile on raw theta power, *F*_(2, 252)_ = 24.75, *p* < 0.001, adj $\eta_{\text{p}}^{2}$ = 0.158, as well as alpha fraction power, *F*_(2, 252)_ = 11.89, *p* < 0.001, adj $\eta_{\text{p}}^{2}$ = 0.079, and theta fraction power, *F*_(2, 252)_ = 66.38, *p* < 0.001, adj $\eta_{\text{p}}^{2}$ = 0.340, but not raw alpha power, *F*_(2, 252)_ = 0.15, *p* = 0.86, adj $\eta_{\text{p}}^{2}$ = 0.007. Also, there were no interactions of measurement time and driving profile, all *F*s < 2.76, all *p*s > 0.06. Further comparisons of the different driving profiles indicated that alpha fraction decreased from simple to complex passages, *F*_(1, 126)_ = 14.36, *p* < 0.001, adj $\eta_{\text{p}}^{2}$ = 0.095, but did not differ in complex and interactive passages, *F*_(1, 126)_ = 0.06, *p* = 0.82, adj $\eta_{\text{p}}^{2}$ = 0.007. Raw theta power increased from simple to complex passages, *F*_(1, 126)_ = 44.27, *p* < 0.001, adj $\eta_{\text{p}}^{2}$ = 0.254, but did not differ in complex and interactive passages, *F*_(1, 126)_ = 0.04; *p* = 0.85, adj $\eta_{\text{p}}^{2}$ = 0.008. Theta fraction power also increased from simple to complex passages, *F*_(1, 126)_ = 52.53, *p* < 0.001, adj $\eta_{\text{p}}^{2}$ = 0.289, and was stronger in interactive than in complex passages, *F*_(1, 126)_ = 10.95, *p* < 0.005, adj $\eta_{\text{p}}^{2}$ = 0.073.

### Correlational Alpha and Theta Power Analysis

**Figure 6:**
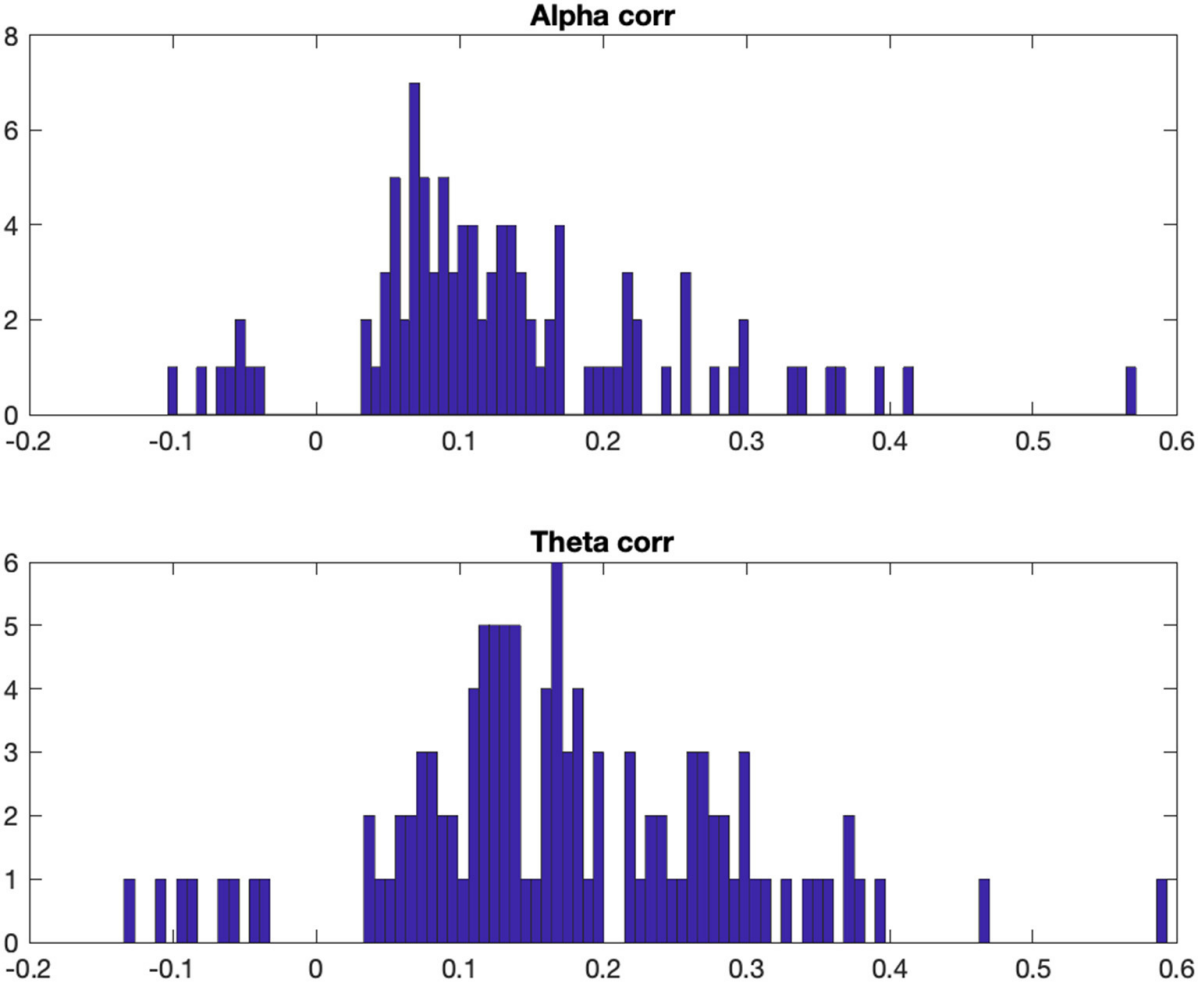
Frequency distribution of individual (significant) Pearson's correlation coefficients for alpha (upper row) and theta (lower row) fraction power measured at MP1 and MP2.

In order to estimate the degree to which alpha and theta fraction power remained stable between the two measurement points at an intraindividual level, correlations have been computed across the entire 30-km driving course (i.e., across the 3,000 10-meter track segments) for each participant. Individual analyses revealed that the correlation coefficients were quite evenly distributed and ranged from low to medium (Figure 6). There were significant positive correlations (*p* < 0.05) in 73.2% of the participants for alpha fraction, *r* = 0.57–0.04, and in 80.3% for theta fraction, *r* = 0.59–0.04. Of these significant positive correlations, 53.8% (alpha fraction) and 68.6% (theta fraction) were in a low range, *r* > 0.1, and 7.5% (alpha fraction) and 13.7% (theta fraction) were in a medium range, *r* > 0.3. Also, significant negative correlations were found in 6.3% of the participants for alpha fraction, *r* = −0.10 to −0.04, and in 6.3% for theta fraction, *r* = −0.13 to −0.04.

### EEG-Based Estimation of Task Load

**Figure 7:**
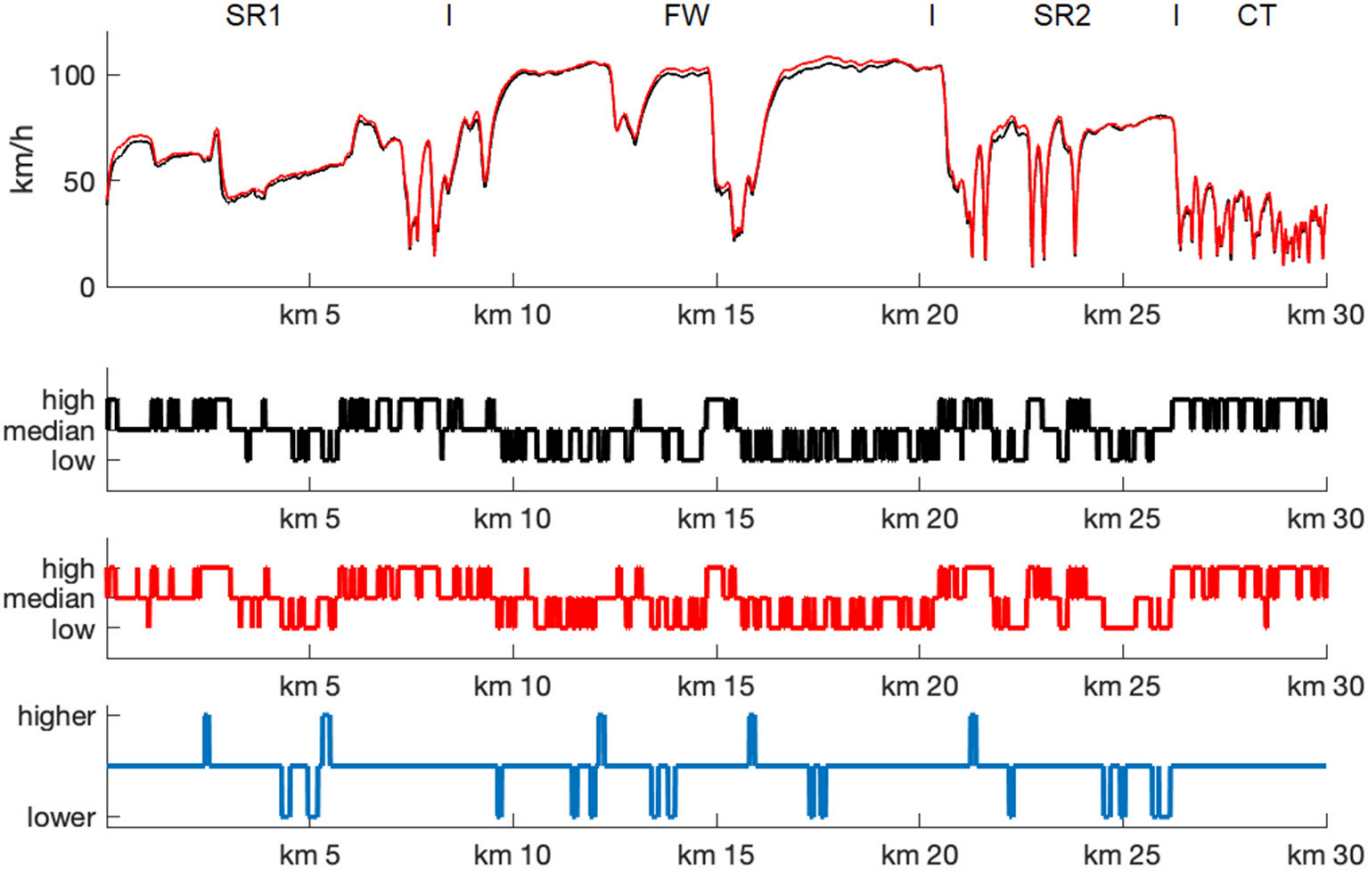
EEG-based estimation of task load. Task load (low, medium, high) as function of driving route for MP1 (black) and MP2 (red), and MP1 – MP2 differences (blue). For each time point individual task load estimations of each participant were assigned to fix waypoints and then averaged separately for 10-meter track segments. For comparison, mean driving speed (upper row) is also shown. SR1, first state road; FW, freeway; SR2, second state road; CT, city traffic.

**Figure 8:**
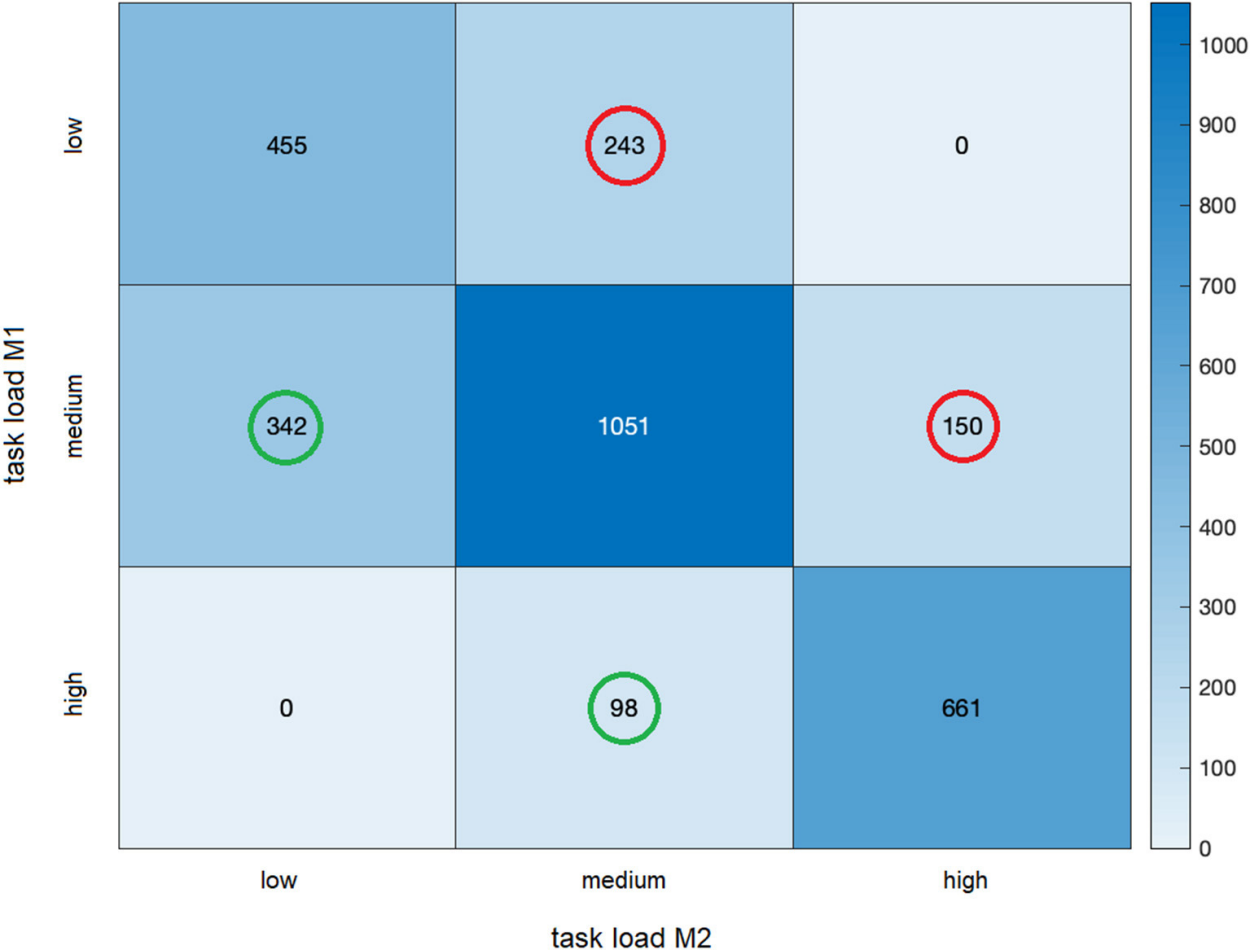
Heatmap of EEG-based estimation of task load at MP1 and MP2. The figure shows the number of road sections that were rated as equally difficult at both measurement times, as well as the number of sections that were rated as easier (outlined in green) or more difficult (outlined in red) in MP2 than in MP1, averaged across all participants. A total of 3,000 (10-meter) track segments were classified as of low, medium, or high task load.

The EEG-based estimation of the track-specific task load revealed a pronounced variance of load ratings along the driving course (Figure 7). High load ratings were mainly found at the beginning of the drive and of the fog passage (at kilometer 3), during the state road sections (SR1 and SR2) as well as during the city traffic drive (CT). Low load ratings were found during the freeway section (FW), apart from passing through a freeway parking area (at kilometer 15). This pattern was overall quite similar at MP1 and MP2, *r* = 0.729. There were, however, some differences in task load ratings: Higher ratings were found at the beginning and the end of the fog passage (at kilometer 3 and 5), while passing through the freeway parking area (at kilometer 15), and at the end of the freeway section. In contrast, lower ratings were found during the fog passage, during the freeway section (FW), and the second state road section (SR2). Overall, of the 3,000 (10-meter) track segments assessed, 72.23% were rated the same in terms of task load, 14.66 % were rated as easier and 13.11% were rated as more difficult. Not a single road section that was rated as easy (difficult) in one of the two measurements was rated as difficult (easy) in the other measurement (Figure 8).

Finally, in order to test to what extent the EEG-based estimates of task load are consistent at the first and second measurement time points at an intraindividual level, correlations were computed across the 3,000 (10-meter) track segments for each participant. There were significant positive correlations in 81.9% of the participants, *r* = 0.44–0.04, of which 61.5% were in a low range, *r* > 0.1, and 4.8% in a medium range, *r* > 0.3. Significant negative correlations occurred in 7.1% of the participants, *r* = −0.11 to −0.04 (all *p* < 0.05).

## Discussion

The aim of the present study was to evaluate the reliability of the cEEGrid technology in a longitudinal investigation of the driving abilities of older adults. Behavioral and electrophysiological parameters of mental load measured while driving in a driving simulator at two time points more than 1 year apart were compared and related to characteristics of the driving course. With a high reliability of the measurement, comparable effects of task difficulty on the EEG parameters should appear (independent of the time of measurement), which should also be related to the behavioral measures. In addition, a high correspondence of the oscillatory measures between the first and the second measurement time should occur on an intra-individual level. The analyses indicated a number of specific effects of measurement time point and driving profile on behavioral driving parameters and brain oscillatory activity that are discussed in detail in the following.

### Driving Parameters: Speed and Steering Wheel Angular Velocity

Overall, the average speed increased while the steering wheel angular velocity decreased from MP1 to MP2. Given that the driving speed in our scenario could be freely chosen by the driver within the maximum speed limits, the increase in driving speed at the second measurement time point could indicate an increase in perceived safety when managing the driving task at a second time. On the one hand, this could result from a higher familiarity with the route. Especially in elderly drivers, a reduction of speed is a frequently observed strategy when driving an unknown route or when the driving situation becomes more complex so that drivers feel unsafe (Trick et al., 2010). In extreme cases, this can lead to dangerous driving situations, for example, if other road users are hindered and forced to make unnecessary and risky overtaking maneuvers. On the other hand, driving speed is usually increased with decreasing workload (Harms, 1986; Verwey and Veltman, 1996), which would also suggest that the second drive was less challenging to the participants than the first one.

This is also supported by the decrease in steering wheel angular velocity as this measure is also considered to be an indicator of task load while driving (e.g., Antin et al., 1990; Verwey and Veltman, 1996). Here, high angular velocities are associated with high load whereas low angular velocities are associated with low load. Accordingly, repeatedly driving the same route would be less stressful than maneuvering on a completely unfamiliar route. It is remarkable that there was more than 1 year between the measurements, which means that the participants seem to have memorized the requirements of the route very well. In addition, long-term learning effects could play a role by which the participants benefit from a more and more experienced anticipation of steering behavior of the car. In a previous driving simulator study, in which younger and older participants had to keep a virtual car on track on a curvy road, we also observed learning effects in form of a decrease in steering variability during the ~1-h drive (Getzmann et al., 2018).

With regard to the reliability of the measurements, it is also remarkable that the influences of the driving profile on driving speed and steering wheel angular velocity did not differ at MP1 and MP2. The driving course was subdivided into simple, complex, and interactive driving profiles, which were related to different levels of task load, based on known factors of mental load in driving situations (Pauzié, 2008; Engström et al., 2017; Rahman et al., 2017). Thus, passages with an undisturbed ride on a free route were rated as of low task load, passages with junctions with turning, roundabouts, and left turns as of medium task load, and interactions with other traffic participants as of high task load (for a critical discussion, see Wascher et al., 2019). The increase in steering angular velocity with increasing task load corresponds well with the assumption that this measure is associated with the demands of driving, which, as expected, is lower for a simple driving profile than for a complex one (involving intersections and traffic lights) as well as interactions with other road users. A limiting factor here could be that the driving profiles were not evenly distributed over the route sections. For example, complex driving profiles (with intersections and give way signs) are more common in the city, while freeway sections are more characterized by simple driving profiles. This might also explain the (unexpectedly) higher driving speed with interactive than complex driving profiles: Interactions with other road users are also common on state roads and freeways (where driving speed is on average higher than in the city), whereas complex driving profiles (and lower driving speed) are more common in the city. An interaction of driving profile and route section can therefore not be completely ruled out. Nevertheless, the replication of this general pattern suggests that overall demands decreased relative to the first test drive, but did not depend on the route profiles passed through.

### EEG Parameters: Alpha and Theta Power

Comparable patterns to the behavioral data were also found in the derived EEG measures: Overall, raw alpha activity increases from the first to the second measurement time point. In addition, and independently of the time of measurement, relative alpha power (power fraction) varied with the driving profiles and was higher at simple compared to complex and interactive passages. In general, decreases in alpha power are usually associated with the allocation of attention (Herrmann and Knight, 2001), while increases in alpha power is assumed to reflect mental fatigue, but also attentional withdrawal and disengagement (Hanslmayr et al., 2012; Wascher et al., 2014, 2016) as typically observed when tasks are perceived as monotonous and boring (Borghini et al., 2014). In the driving context, increased alpha power has thus been observed during monotonous driving situations, probably reflecting periods of inattention and mind-wandering (Lin et al., 2016). Assuming increases in alpha activity to be associated with reduced attentional engagement, the present findings would argue for a withdrawal of attentional resources, both in longitudinal and route-related terms: The participants seemed to pay less attention to the driving task when they drove the same route for a second time. However, they continued to flexibly adapt their mental resources to the task demands and increased their attention when the traffic situation became more complex. Interestingly, effects of driving profile were only found on alpha fraction power, but not raw alpha power. This discrepancy could be due to a relatively high power in oscillatory activity in low frequency bands (as has been observed in Wascher et al., 2019), which could have masked experimental effects in the higher frequency alpha band. In line with this assumption, it has been shown that weak effects in higher frequency bands tend to become evident in relative power measures rather than in absolute (raw) measures, where low-frequency power is dominant (Labounek et al., 2019).

Activity in theta power is generally associated with cognitive control (Cavanagh and Frank, 2014; Cavanagh and Shackman, 2015) and typically increased with higher workload (Wilson and Hankins, 1994; Gevins et al., 1997) and task demands (Lal and Craig, 2001; Jensen and Tesche, 2002; Onton et al., 2005; Borghini et al., 2014). Theta power also increases with higher task engagement (Yamada, 1998; Onton et al., 2005) and with the effort to keep task performance high (Wascher et al., 2014; Arnau et al., 2017). In line with this assumption, both raw and relative (fractional) theta power values were increased in the present driving task at more complex route sections, such as at the beginning of the fog passage and during city driving. However, independently of these demand-related modulations, there was rather an (albeit not significant) increase in raw theta power at the second measurement time point (cf. Figure 4), suggesting that the task demands and/or task engagement increased (or at least did not differ) at MP2 compared to MP1.

One could argue that the theta power findings partly contradict the interpretation of the alpha power, suggesting a decrease in task engagement. Selection effects in the way that drivers with low task engagement left the study after MP1 and did not participate in MP2 can be excluded, as the same drivers were examined at both measurement time points. A more plausible interpretation could be that the participants had a higher motivation to perform well in the driving task, perhaps even better than at the first time. Given that the current study is designed to detect age-related deteriorations in driving ability, the participants' motivation to counteract these by increasing effort may be particularly pronounced, as reflected by undiminished theta activity. This interpretation is supported by findings of a previous study on age-related differences in pro-active driving behavior (Getzmann et al., 2018): Better performance in proactive driving (i.e., more alert steering behavior, better anticipation and active use of driving-relevant information and more proactive planning of driving manoeuvers) was associated with increased mental effort in the older group, as reflected by higher theta power. Moreover, only in the older group a relationship between steering variability and theta power was found, with better steering performance being associated with higher theta power. Taken together, the EEG findings suggest that the drivers were more relaxed, but remained motivated to perform the driving task well at the second time.

Another relevant aspect to be discussed here are task-specific differences between alpha and theta activity, which are also reflected in differences in the brain areas over which they are usually derived. While alpha power is most prominent over occipital-parietal areas of visual cortex, theta power is measured over frontal areas associated with higher cognitive executive functions (for review, Klimesch, 1999). In a realistic driving task, in which complex and monotonous driving passages alternate, and in which multiple subtasks such as visual information uptaking and processing, attention allocation, spatial navigation have to be performed, alpha and theta activity should therefore be differently involved (Di Flumeri et al., 2018; Puma et al., 2018; Wang et al., 2018). In particular, alpha activity (i.e., its suppression) seems to be rather associated with task engagement, while theta activity seems to be associated with task workload (Berka et al., 2007; Wang et al., 2018). This could explain, for example, differences in the dependence of alpha and theta power on the driving profile. For example, the track-based analysis indicated an increase in theta activity at the beginning of the second state road section at kilometer 21 (which was characterized by demanding passages), which was not accompanied by a suppression of alpha activity (cf. Figure 4). Also, theta fraction power was higher in interactive than in complex driving profiles, whereas alpha fraction power did not differ in either condition. This could suggest that interaction with other road users and driving on a demanding but empty route account for differences in task load, while task engagement is hardly affected.

A limiting factor which complicates the interpretation of the results, and which has to be noted here is the reduced spatial resolution of the cEEGrid technology. The electrodes are located largely over temporal areas, making a clear differentiation of oscillatory activity into frontal and parieto-occipital parts difficult. Traditionally, theta activity is derived over frontal areas and alpha activity over posterior areas, where the power in these frequency bands is usually most prominent (for review, Klimesch, 1999). Thus, only very few studies have considered theta and alpha activity measured over temporal areas as potential indices of mental workload and task engagement. In studies on simulated driving (Diaz-Piedra et al., 2020) and multi-tasking (Puma et al., 2018), workload-induced modulations of theta activity (with higher workload being associated with higher theta activity) were not only observed over frontal and occipital regions, but also over temporal regions. A combined EEG-fMRI study showed that workload-induced modulations of theta activity were most pronounced over frontal and posterior areas (Sammer et al., 2007). However, an additional EEG-constrained fMRI analysis revealed that the generators of these effects were not primarily localized frontally, but form a network including temporal and hippocampal hemodynamic activation, cingulate activation, frontal superior, and cerebellar activation. The authors thus concluded that theta band activity reflects a binding process of widely distributed cortical areas, which all contribute to the EEG activity derived at the scalp. The same could be true for alpha band activity, which appears to reflect a network-binding mechanism, supporting the interplay within thalamo-cortical networks relevant for sensory gating and the control of vigilance and attention (Lopes da Silva, 2013; for review, Nishida et al., 2015). Significant effects of task difficulty on alpha power (with easier task conditions being associated with larger power) have been observed over temporal areas (Brookings et al., 1996), while other studies failed to find effects of performance (Çiçek and Nalçaci, 2001) and relaxation (Scholz et al., 2018) on alpha activity over temporal areas, which were observed over parietal areas. Thus, it appears that theta effects could be more reliably derived over temporal areas than alpha effects. This could also explain why in the present study (as well as in our previous study, Wascher et al., 2019) discrepancies between raw and fractional power occurred in the alpha band, but not in the theta band: Given that fractional power was corrected for total power in the signal, alpha effects could be more pronounced (independent of their topography) in fractional power. However, since no conventional multi-channel EEG cap has been employed here for a direct comparison of the signals measured with cEEGrids, especially the interpretation of the alpha activity should be treated with caution.

### Test-Retest Reliability Considerations

Two different approaches have been chosen to determine the retest reliability of the EEG results, first a task-load related analysis, investigating whether the EEG measures at both measurement points depended on the driving profile in the same way, and second an (intra-individual) correlational analysis, comparing the EEG measures along the route (subdivided into 3,000 10-m track segments) at MP1 and MP2 separately for each subject. The task-load related analysis showed a high correspondence of the EEG patterns between the two measurement times across all participants: That is, independent of the measurement time, challenging traffic situations are accompanied by a reduction of alpha and an increase of theta (e.g., as can be seen at the beginning of the city drive), whereas monotonous traffic situations (e.g., the foggy passage or the undisturbed highway drive) showed the opposite pattern. Thus, the effect of driving profile on alpha and theta activity was reliably found at both measurement times, indicating a high reliability of the measurement, especially for fraction values.

The same is true for the high correspondence in the estimation of task load from the alpha and theta values between the two measurement time points. The track-based analysis indicated that passages that were estimated to be easy (or hard) at MP1 were also easy (or hard) at MP2. In particular, averaged across all participants, the analysis showed that not a single road section that was rated as easy (difficult) in MP1 or MP2 was rated as difficult (easy) in the other measurement (cf. heatmap in Figure 8), which indicates a reliable estimation of task load. On the other hand, this also means that road sections overall were not estimated to be easier at MP2. Thus, a higher familiarity with the route (suggested by a higher average speed and lower steering wheel angular velocity) was not associated with a reduced difficulty (estimated from the alpha and theta ratio). In other words: a difficult passage (associated with high theta and low alpha activity) may well be passed more quickly due to familiarity with the route, without it becoming less challenging. Still, a few changes emerged that can be plausibly explained (as can the driving parameters). For example, the patterns of alpha/theta values at the second measurement time point indicate an increased task load at the beginning and end of the fog passage, whereas during the fog passage the task load was estimated to be lower. Both effects can be explained by an increasing familiarity of the participants with the route. This interpretation is in line with the so-called “route-familiarity effect,” in which greater route familiarity can lead to increased inattention and mind-wandering and, as a consequence, to driving impairments (e.g., Martens and Fox, 2007; Yanko and Spalek, 2013). The same was true for undisturbed country road passages, which appeared to be driven with a higher routine and lower task load. This and the overall high correspondence of the EEG patterns with the behavioral data suggest high content test-retest reliability of the cEEGrids technology used for the sample of participants as a whole.

On an intraindividual level, significant positive correlations were found for most of the participants, both for alpha and theta activity as well as for the derived EEG-based estimation of task load. Participants who showed a high alpha or theta activity at the first measurement time and a high mental workload did so again at the second measurement time, which indicates a certain degree of temporal stability of the measurements. However, it has to be noted that the vast majority of the individual correlations were (although mostly highly significant) in a low range. Usually, higher reliabilities are found in more structured EEG conditions (McEvoy et al., 2000), i.e., in demanding cognitive tasks, since fluctuations in cognitively engaging tasks are generally lower. At least in complex road sections and in interaction with other road users, an increased cognitive load can also be assumed.

Regarding an individual (possibly diagnostic) evaluation, the small correlations make the interpretation in terms of a change from MP 1 to MP 2 difficult. The same applies to the prediction of future values based on previous values. In addition, it is remarkable that some (few) participants also showed negative correlations, suggesting that the pattern of oscillatory power over the driving distance has (at least partly) reversed. This could indicate a change in the mental resources that some subjects invested in the driving task, with a high task engagement at MP1 changing to an attentional disengagement at MP2 (or vice versa). In this context, it should be pointed out that the data come from an ongoing study on the development of traffic safety parameters in older drivers, and that changes in mental abilities are to be expected in the age range considered.

In summary, however, it must be stated that the correlations within the participants are rather low, i.e., that the alpha/theta activity in track segments at MP1 is poorly associated with the alpha/theta activity in the same segment at MP2. This suggests high fluctuations in oscillatory activity between measurement time points that are not related to the task load of the track segments themselves. It is difficult to assess whether this is due, for example, to transient fluctuations in mental states like alertness and vigilance during the drive, or changes within participants over the relatively long time period between MP1 and MP2, or demonstrates limitations of the EEG methodology used. Further insights may be provided by the investigation of possible correlations between changes in individual driving performance (and its changes over time) and EEG parameters, which are planned at a later stage of the still ongoing project. The age range of the test group, which is clearly not representative for the entire population of drivers, may also be a potentially limiting factor with regard to the generalizability of the results. Age-related decreases in cognitive performance as well as increases in interindividual variation both can lead to a conflict with the determination of the reliability of the EEG method. Another problem specific to the cEEGrid technology is that the electrodes on older skin, which is often drier and more wrinkled, may have increased resistances, resulting in poorer and fluctuating conduction of the EEG. Future comparative studies with younger subjects therefore seem appropriate.

## Conclusions

Taken together, the present test-retest analysis demonstrated changes in behavioral and brain oscillatory parameters between the first and second measurement time point across all participants, which can be characterized by an increase in driving speed and decrease in steering angular velocity as well as an increase in alpha power, while theta power remained rather stable. These changes suggest a reduced overall task load which appears plausible with regards to learning and memory effects. At both measurement points, the EEG parameters (like the behavioral parameters) were similarly modulated by track difficulty and—as a consequence—task demands, indicating a high reliability and ecological validity of the EEG application via cEEGrid technology. At the intra-individual level, positive correlations of the oscillatory measures and its dependence on track difficulty were found in the majority of the participants. On the other hand, intra-individual correlations were (although significant) rather low, raising the question of the individual-diagnostic value of the chosen method. Further analysis of the reasons why some participants showed significant differences compared to the first measurement will be necessary to determine if this was due to the EEG recording or if the causes may be found in the participants themselves (e.g., cognitive decline). However, in the context of task-related EEG parameters which maps changes in cognitive states related to, for example, task difficulty, the reliability and ecological validity of cEEGrid electrodes appear satisfactory. Overall and in combination with the findings of our previous study (Wascher et al., 2019), the results provide further evidence for the usability of portable low-density EEG methods like cEEGrids as an alternative to conventional lab-based recording systems for mapping mental processes in natural environments.

## Data Availability Statement

The datasets presented in this article are not readily available because the funder of this study does not allow sharing of data. Requests to access the datasets should be directed to Stephan Getzmann, getzmann@ifado.de.

## Ethics Statement

The studies involving human participants were reviewed and approved by Local Ethics Committee of the Leibniz Research Centre for Working Environment and Human Factors. The patients/participants provided their written informed consent to participate in this study.

## Author Contributions

SG, GR, MK, and EW were responsible for the design of the study. EW analyzed the data. JR was involved in data acquisition and provided data and analyses of a pilot-study. SG wrote a first version of the manuscript. All authors contributed to the completion of the final version of the manuscript.

## Funding

This report is based on parts of the research project carried out at the request of the Federal Ministry of Transport and Digital Infrastructure, represented by the Federal Highway Research Institute, under research project No. 82.0649/2016. The authors are solely responsible for the content. The publication of this article was funded by the Open Access Fund of the Leibniz Association.

## Conflict of Interest

The authors declare that the research was conducted in the absence of any commercial or financial relationships that could be construed as a potential conflict of interest.

## Publisher's Note

All claims expressed in this article are solely those of the authors and do not necessarily represent those of their affiliated organizations, or those of the publisher, the editors and the reviewers. Any product that may be evaluated in this article, or claim that may be made by its manufacturer, is not guaranteed or endorsed by the publisher.
